# A systematic review of economic evaluations of cardiac rehabilitation

**DOI:** 10.1186/1472-6963-12-243

**Published:** 2012-08-08

**Authors:** Wai Pong Wong, Jun Feng, Keng Ho Pwee, Jeremy Lim

**Affiliations:** 1 Academic Programmes Division, Singapore Institute of Technology, 25 North Bridge Road, Singapore, Singapore; 2 Primary & Community Care Division, Ministry of Health, 16 College Road, Singapore, Singapore; 3 OptumInsight, 370-372 Norton Street, New South Wales, Australia; 4 Performance and Technology Assessment Division, Ministry of Health, College Road, Singapore, Singapore; 5 Fortis Healthcare Singapore, 180 Clemenceau Avenue, Singapore, Singapore

## Abstract

**Background:**

Cardiac rehabilitation (CR), a multidisciplinary program consisting of exercise, risk factor modification and psychosocial intervention, forms an integral part of managing patients after myocardial infarction (MI), revascularization surgery and percutaneous coronary interventions, as well as patients with heart failure (HF). This systematic review seeks to examine the cost-effectiveness of CR for patients with MI or HF and inform policy makers in Singapore on published cost-effectiveness studies on CR.

**Methods:**

Electronic databases (EMBASE, MEDLINE, NHS EED, PEDro, CINAHL) were searched from inception to May 2010 for published economic studies. Additional references were identified through searching bibliographies of included studies. Two independent reviewers selected eligible publications based on the inclusion/exclusion criteria. Quality assessment of economic evaluations was undertaken using Drummond’s checklist.

**Results:**

A total of 22 articles were selected for review. However five articles were further excluded because they were cost-minimization analyses, whilst one included patients with stroke. Of the final 16 articles, one article addressed both centre-based cardiac rehabilitation versus no rehabilitation, as well as home-based cardiac rehabilitation versus no rehabilitation. Therefore, nine studies compared cost-effectiveness between centre-based supervised CR and no CR; three studies examined that between centre- and home based CR; one between inpatient and outpatient CR; and four between home-based CR and no CR. These studies were characterized by differences in the study perspectives, economic study designs and time frames, as well as variability in clinical data and assumptions made on costs. Overall, the studies suggested that: (1) supervised centre-based CR was highly cost-effective and the dominant strategy when compared to no CR; (2) home-based CR was no different from centre-based CR; (3) no difference existed between inpatient and outpatient CR; and (4) home-based programs were generally cost-saving compared to no CR.

**Conclusions:**

Overall, all the studies supported the implementation of CR for MI and HF. However, comparison across studies highlighted wide variability of CR program design and delivery. Policy makers need to exercise caution when generalizing these findings to the Singapore context.

## Background

Cardiovascular diseases are a major cause of mortality and morbidity, contributing to about 30% of all-cause mortality and 10% of the total disability-adjusted life-years globally [1]. Although advances in medical therapy and revascularization surgery have improved outcome, reducing risk factors associated with cardiovascular diseases remains an important strategy in lowering the global burden of disease [2]. Risk factor management is a core component of cardiac rehabilitation, which in turn forms part of the overall management of patients with cardiovascular diseases such as coronary artery disease or chronic heart failure [3,4].

Besides risk factor management (specifically control or reduction of lipids, blood pressure, body weight, diabetes mellitus and cigarette smoking), the other core components of cardiac rehabilitation include nutritional and physical activity counseling, psychosocial interventions and exercise training [4]. In particular, exercise training is often the component being examined under the umbrella term ‘cardiac rehabilitation’, likely because of its duration and therefore the cost of the program [5].

Several systematic reviews over the past three decades have consistently demonstrated cardio-protective effects of exercise-based cardiac rehabilitation programs [6-9]. Exercise-based cardiac rehabilitation, compared to usual care, reduces all-cause mortality by 20% (95% confidence interval, CI: 7%, 32%) and cardiac mortality 26% (95% CI: 4%, 39%) [8]. Risk factors such as total cholesterol, triglycerides, systolic blood pressure and self-reported smoking habits were also significantly reduced [8]. The pooled sample size for the most recent systematic review was 8,940, most of whom had undergone at least two months of cardiac rehabilitation under supervision of professional exercise personnel [8]. This implies great involvement of economic cost in the delivery of cardiac rehabilitation.

Economic evaluation of cardiac rehabilitation has been reported since the 1980s. A systematic review of economic evaluation studies on cardiac rehabilitation, which identified 15 studies, was reported in 2005. Based on studies published between 1985 to 2004, supervised cardiac rehabilitation, compared to usual care, resulted in USD2,193 to USD28,193 per life year gained, and USD668 to USD16,118 per quality-adjusted life years(monetary values were 2004 US dollars) [10]. Most of the studies reviewed up to 2004 were based on prospective randomized controlled trials conducted much earlier than their published dates. Over the past five years, more economic evaluation studies emerged. These studies might involve patients who have undergone more recent medical therapies for coronary artery disease and chronic heart failure. Recent studies have also focused on comparisons among different modes of delivery of cardiac rehabilitation, such as programs that were outpatient-, inpatient- as well as home-based. Therefore it is timely to systematically review and summarize the evidence on cost-effectiveness of cardiac rehabilitation.

The overall objective of the current systematic review was to describe and summarize published economic evaluations of cardiac rehabilitation for comparing the cost-effectiveness of different modes of delivery of cardiac rehabilitation. The specific aims were to compare the following modes of delivery:

(a) supervised cardiac rehabilitation versus no cardiac rehabilitation,

(b) supervised versus home-based cardiac rehabilitation,

(c) inpatient (not Phase I ward program, but residential Phase II program) versus outpatient cardiac rehabilitation, and

(d) home-based cardiac rehabilitation versus no cardiac rehabilitation.

In this review, cardiac rehabilitation is considered as consisting of at least exercise training sessions, as this is usually the component studied as well as being the main cost driver of cardiac rehabilitation programs.

## Methods

### Search strategy

Prior to developing the search strategy, “PICO” statements were used to address the specific aims of the systematic review (Table 1). The electronic databases of EMBASE, MEDLINE, NHS EED, PEDro and CINAHL was searched using the text word terms of ‘economic evaluation’, ‘cost’, ‘cost-effectiveness’, ‘cost-benefit’ or ‘cost-utility’, and ‘cardiac rehabilitation’ up to May 2010. Hand searches of bibliographies of each reference followed to identify any additional publications. Unpublished or grey literature was not included.

**Table 1:** PICO statements used to develop the search to address the four different modes of delivery

***PICO***	***(a)***	***(b)***	***(c )***	***(d)***
Population	Patients diagnosed with acute MI or chronic HF	Patients diagnosed with acute MI or chronic HF	Patients diagnosed with acute MI or chronic HF	Patients diagnosed with acute MI or chronic HF
Intervention	Supervised outpatient CR	Supervised outpatient CR	Supervised outpatient CR	Home-based CR
Comparison	Usual/standard care (i.e., no CR)	Home-based CR	Supervised inpatient CR	Usual/standard care (i.e., no CR)
Outcome	Cost-effectiveness	Cost-effectiveness	Cost-effectiveness	Cost-effectiveness

MI, myocardial infarction; HF, heart failure; CR, cardiac rehabilitation.

### Selection criteria

For the purpose of this review, the inclusion criteria were as follows:

Economic evaluation study design that was either prospective alongside a clinical trial or based on modeling;

Adult patients diagnosed with acute myocardial infarction (including post-infarction and after revascularization surgery or percutaneous coronary intervention for infarct), or chronic heart failure;

Intervention that included exercise-based cardiac rehabilitation;

Study with at least one of four comparators listed in Table 1;

Outcomes included either cost-effectiveness, cost-utility or cost-benefit analysis. Cost-minimization analysis was not included.

Articles were excluded if one of the elements of PICO (Table 1) was not met.

### Quality assessment

The 10-item Drummond checklist was used to assess the methodological quality of the economic evaluation studies [11]. If the study met any of these 10 items, it would be considered as ‘Yes’, otherwise ‘No’ or ‘Cannot tell’ (Additional file 1: Appendix 1). The Drummond checklist provides a global assessment of the quality of evidence, but does not form the basis for accepting or rejecting articles.

### Data abstraction

Two independent reviewers (JF and WPW) selected eligible publications initially based on titles and abstracts. Potentially relevant articles were abstracted using standardized data abstraction form. This form was also used for data synthesis. Any disagreement between the reviewers was resolved by consultation with a third reviewer (KHP).

## Results

### Synthesis

**Figure 1:**
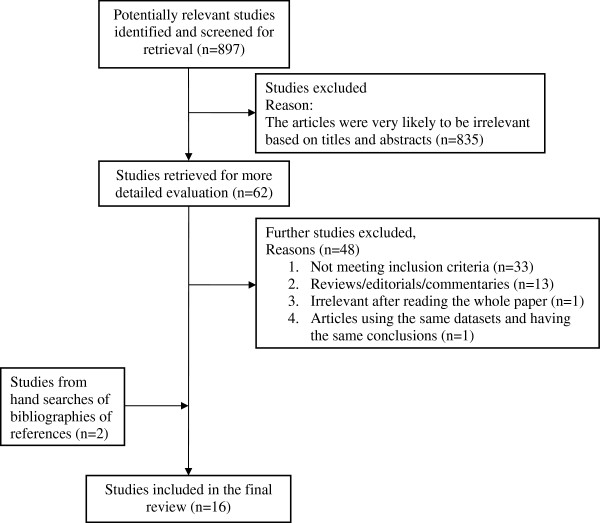
Flow of included studies.

A total of 896 citations were retrieved based on the search strategy. Of the 62 articles retrieved for more detailed evaluation, only 20 were included. An additional two references from hand searches yielded a total of 22 [12-33] articles for review. Five articles were concerned with cost-minimization analyses, whilst one article included data from patients with stroke in its economic modelling. The remaining 16 articles included one article that addressed both centre-based cardiac rehabilitation versus no rehabilitation, and home-based cardiac rehabilitation versus no rehabilitation. Among the six foreign-language articles, three did not meet the inclusion criteria, two were editorials or commentaries and one was deemed irrelevant after reading through the full article. Therefore, only 16 articles were included. Figure 1 describes the reasons for not including the articles.

The majority of the articles clearly stated that they included patients after acute myocardial infarction, following revascularization surgery or percutaneous coronary interventions, or who had a diagnosis of chronic heart failure, and therefore satisfied the selection criterion on diagnosis. Two articles employed the same sample for analyses, involving patients with coronary artery disease, defined as acute myocardial infarction or angina pectoris [25,27]. One study included patients with coronary artery disease, defined as acute myocardial infarction (75% and 79% respectively in both groups) or post-angioplasty (25% and 21% respectively) [32]. In another study, patients after acute myocardial infarction accounted for 39%, chronic heart failure 22%, angina 45%, arrhythimias 59%, and valvular disease 26% [30].

The quality of these articles varied. Articles fulfilled two to nine items on the Drummond checklist, with none of them meeting all the items (Additional file 1: Appendix 1). Table 2 summarizes the economic evaluations reported in these studies (details in Additional file 2: Appendix 2). Five of these studies were cost-minimization analysis, and therefore were strictly not considered as full economic evaluations [11].

**Table 2:** Summary of economic evaluations comparing supervised centre-based cardiac rehabilitation (CR) vs no cardiac rehabilitation (No-CR)

**Author (Year)**	**Perspective**	**Patient populations**	**Study type**	**Currency, price year**	**Findings**
Levin et al. (1991)	Societal	N = 305 after MI	CCA	Swedish kroner, ?year	CR: SEK73,500 less per patient
Ades et al. 1992	Patients/ payers	N = 580 after MI/CABG	CCA	US dollars, 1991	CR: $739 less in hospitalization costs per patient
Oldridge et al. (1993)	Societal	N = 201 after MI	CUA/ modelling	US dollars, 1991	CR: $21,800 per life-year gained; $9,200 per QALY gained at 1 year
Ades et al. (1997)	Patients/ Payers	Not applicable	Economic modeling	US dollars, 1995	CR: $4,950 per year of life saved
Georgiou et al. (2001)	Societal	N = 99 with HF	CEA	US dollars, 1999	ICER = $1,773 per life year saved in favour of CR
Marchionni et al. (2003)	Government or care providers	N = 158 with MI	CCA	US dollars, 2000	CR: $21,298 per patient *vs* $12,433 per patient in No-CR group
Yu et al. (2004)	Government	N = 204 after MI or PCI	CUA	US dollars, ?year	ICUR = $650 per QALY in favour of CR
Huang et al. (2008)	Government	N = 4,324 after CABG	CEA	US dollars, 1998	ICER = 13,887 per year of life saved in favour of CR
Dendale et al. (2008)	Health care payers	N = 213 after PCI	CEA	Euro, ?year	CR: 4,862€ per patient and 5,498€ per patient in No-CR group

MI, myocardial infarction. HF, heart failure. CABG, coronary artery bypass graft surgery. PCI, percutaneous coronary intervention. CCA, cost-consequences analysis. CEA, cost-effectiveness analysis. CUA, cost-utility analysis. QALY, quality-adjusted life-years. ICER, incremental cost-effectiveness ratio. ICUR, incremental cost-utility ratio.

### Supervised (or centre-based) cardiac rehabilitation versus no cardiac rehabilitation (usual/standard care)

A total of nine studies examined the cost-effectiveness of supervised centre-based cardiac rehabilitation compared to no cardiac rehabilitation. Most of the economic studies were conducted prospectively alongside randomized controlled trials (RCT) [14,16-18], whilst three studies used modeling to derive long-term cost-effectiveness [14-16]. All the studies suggested that cardiac rehabilitation supervised at a facility compared to no cardiac rehabilitation was cost-saving [12,13,20], cost-effective [14,17,19] and a dominant strategy (that is, less cost, more effective) [16,18]. However, cost-effectiveness could become less because of escalating medical costs, as demonstrated by one modeling study [15].

### Supervised (or centre-based) versus home-based cardiac rehabilitation

Among 10 studies, six yielded no significant differences in the clinical outcome measures, and were therefore technically considered cost-minimization analyses [17,21,23,24,28,33]. Of the remaining four studies, one study employed economic modelling in cost-effectiveness analysis, using data from patients with cardiovascular disease, defined as ‘self-report of previous heart attack, stroke, or other heart disease’ [22]. Among the three included studies (Table 3), one showed that centre-based strategy was dominant compared to home-based rehabilitation [27], whilst two studies demonstrated no difference in cost-effectiveness [25,26]. In one of the cost-minimization analyses, cost to the government (taxation-based health care system) was greater with home-based program than centre-based program, likely due to frequent home visits by hospital staff [28]. The definition of ‘home-based program’ varied among the three studies, involving combinations of home visits [25,26] and decreased frequency of centre-based rehabilitation attendances [25,27].

**Table 3:** Summary of economic evaluations comparing supervised centre-based cardiac rehabilitation (CR) vs home-based cardiac rehabilitation (HCR)*

**Author (Year)**	**Perspective**	**Patient populations**	**Study type**	**Currency, price year**	**Findings**
Reid et al. (2005)	Health system	N = 392 CAD	CCA	US dollars, 2004	HCR: $5,267 per patient
					CR: $5,132 per patient; no difference
Taylor et al. (2007)	Societal	N = 80 MI	CUA	Sterling pounds, 2002-3	ICUR = −£644 per QALY in favour of CR but not significantly different
Papadakis et al. (2008)	Health system	N = 392 CAD	CUA	US dollars, 2004	ICUR = $11,400 per QALY in favour of CR

MI, myocardial infarction. CAD, coronary artery disease. CCA, cost-consequences analysis. CUA, cost-utility analysis. QALY, quality-adjusted life-years. ICUR, incremental cost-utility ratio. * cost-minimization analyses were not included in this table (refer to Additional file 2: Appendix 2 for details).

### Inpatient versus outpatient cardiac rehabilitation

The only study that evaluated cost-effectiveness between inpatient and outpatient cardiac rehabilitation demonstrated no significant difference [29] (Table 4).

**Table 4:** Summary of economic evaluations comparing supervised centre-based inpatient cardiac rehabilitation (ICR) vs supervised centre-based outpatient cardiac rehabilitation (CR)

**Author (Year)**	**Perspective**	**Patient populations**	**Study type**	**Currency, price year**	**Findings**
Schweikert et al. (2009)	Societal	N = 147 MI	CEA/CUA	Euro, 2006	ICER = −165,276€ per QALY in favour of CR, although no significant

MI, myocardial infarction. CEA, cost-effectiveness analysis. CUA, cost-utility analysis. QALY, quality-adjusted life-years. ICER, incremental cost-effectiveness ratio.

**Table 5:** Summary of economic evaluations comparing home-based cardiac rehabilitation (HCR) and no cardiac rehabilitation (No-CR)

**Author (Year)**	**Perspective**	**Patient populations**	**Study type**	**Currency, price year**	**Findings**
Wheeler et al. (2003)	Patients/ payers	N = 452 women with MI, HF, etc.	CCA	US dollars, 2000	HCR: 49% lower inpatient cost; 46% fewer inpatient days
Southard et al. (2003)	Patients	N = 104 MI, CABG, HF	CCA/ CBA	US dollars, ?year	HCR: cost $1,418 less with 213% return on investment
Marchionni et al. (2003)	Government or care providers	N = 153 MI	CCA	US dollars, 2000	HCR: $13,246 per patient; better outcomes
					No-CR: $12,433 per patient
Salvetti et al. (2008)	Health providers	N = 39 CAD	CCA	US dollars, ?year	HCR: $502.71 more per patient

MI, myocardial infarction. HF, heart failure. CABG, coronary artery bypass graft surgery. CAD, coronary artery disease. CCA, cost-consequences analysis. CUA, cost-utility analysis. CBA, cost-benefit analysis.

### Home-based cardiac rehabilitation versus no cardiac rehabilitation (usual/standard care)

Four studies considered the cost-effectiveness of home-based program compared to no cardiac rehabilitation program. Home-based program was considered affordable [32] and more cost-effective than no cardiac rehabilitation [17]. Two studies demonstrated cost-savings [30,31] with home-based program. Of interest was the internet-based program by one of these studies [31].

## Discussion

This systematic review summarizes the cost-effectiveness of cardiac rehabilitation compared to no cardiac rehabilitation, for patients after myocardial infarction, revascularization surgery or percutaneous coronary interventions, as well as those with chronic heart failure. Pooling of results is not possible given the heterogeneity in perspectives, health systems, study designs, details of cardiac rehabilitation interventions and types of patients that exist among the studies included in this review. However, we contend that these studies provide sufficient evidence for policy development concerning cardiac rehabilitation.

Inclusion of a supervised outpatient cardiac rehabilitation program is clearly more cost-effective than not including cardiac rehabilitation program (“usual or standard care”) into the overall management of patients after myocardial infarction or those with chronic heart failure. The centre-based programs consisted of exercise-based sessions, three times a week, over a period of 8 to 12 weeks. In addition, risk factor management and other multidisciplinary input were included in half of these studies.

Four of the nine studies were economic evaluations alongside prospective randomized controlled trials [14,16-18]. Two of these studies examined cost-effectiveness from the societal perspective within differing health systems [14,16]. In the Canadian health system, cardiac rehabilitation compared to usual care had an incremental cost-effectiveness of USD9,200 per quality-adjusted life-year (QALY) gained as well as USD21,800 per life-year saved (1991 US dollars) at 12 months [14]. In the United States’ private health care system, cardiac rehabilitation was the dominant strategy compared to no cardiac rehabilitation with $1,773 (2001 US dollars) per life-year saved at 14 months [16]. Recent economic studies with non-randomized group allocation designs corroborated this observation, for example, an incremental cost-effectiveness ratio of $13,887 per life-year saved (1998 US dollars) was estimated based on Medicare expenditures for American patients undergoing cardiac rehabilitation; these patients had concomitant end-stage renal failure requiring haemodialysis and post-coronary artery bypass graft surgery [19]. In Belgian patients following percutaneous coronary interventions, cardiac rehabilitation led to reduced hospitalization and revascularization surgery, and subsequently cost (published in 2008, in euros) [20]. One economic modeling study suggested that cost savings could become less over the years as a result of rising health care costs [15]. Much of the cost escalation could be attributed to the high costs of cardiac investigations and surgery, in addition to the personnel-intense multidisciplinary cardiac rehabilitation program [26]. Therefore, home-based programs have been touted as a cost-effective alternative.

Comparisons between home-based and centre-based programs were predominantly cost-minimization studies [17,21,23,24,28,33]. In all these studies, the consequences of both alternatives were equivalent, so the authors sought to only compare their costs. Despite different settings, these studies consistently showed that home- and centre-based cardiac rehabilitation to be similar in cost.

Although all 13 studies (including cost-minimization analyses) on home-based cardiac rehabilitation, compared to either supervised centre-based programs or no cardiac rehabilitation, have demonstrated home-based model to be cost-effective or cost-saving, the contents of the home-based programs varied widely. The contents of home-based program ranged from actual exercise sessions at home [21,22,33], frequent home visits by case managers and physicians [24-26,28], to reduced or more spaced-out attendances at the centre [17,23,25,27]. Exercise participation has to be regular to be effective. The option of reduced or spaced-out attendances at the centre is primarily to encourage the patients to continue with the exercises at home, whilst providing opportunity to return to the centre for reinforcement, monitoring and evaluation. One program was internet-based, requiring computer literacy, internet access at home and frequent log-ons to the web site to update on completion of exercises [31].

Early studies (before 2005) tended to demonstrate that home-based programs were more cost-effective and cost-saving than centre-based ones [17,21-24,33]. Sensitivity analyses in some of these studies have shown no change to the conclusion despite taking the worst-case scenario[17] or varying variables such as costs [24], readmission rates [24], patients’ travelling time [24], exercise adherence [22] and discounting rates [22,24]. Home-based programs in these studies were varied, including program with reduced sessions at the centre to exercise program conducted entirely at home with or without frequent home visits by health care professionals (see Table 3). However, the recent studies have shown otherwise [25-28]. Three recent studies demonstrated no significant difference in the cost-effectiveness of centre- *versus* home-based programs [25,26,28]. In one study, sensitivity analyses by taking the upper estimate of UK hospital rehabilitation costs did not alter the conclusion, because cardiac-related costs far exceeded rehabilitation costs [26]. All three economic evaluations were conducted alongside randomized controlled trials, within a taxation-based health care system (Canada and UK) and involved multiple home visits by health care professionals (case managers, physicians and rehab nurses). One recent study demonstrated greater quality adjusted life-year gained among participants in centre-based program than those in home-based program [27]. The “home-based” program in this study was 33 cardiac rehabilitation sessions spread across 12 months, whereas the centre-based program was the same 33 sessions conducted over 3 months. Interestingly, these authors found that the spread-out program was more cost-effective among women whilst the centre-based program was more cost-effective among men [27]. Thus, the cost-effectiveness of the so-called home-based cardiac rehabilitation program depends heavily upon its contents as well as patient profiles. Policy decision makers, and payers or purchasers of cardiac rehabilitation services, should take into consideration of the model of home-based programs when considering resource allocation. The use of information and communication technology and internet-based programs should be explored, and therefore further studies could compare internet- *versus* centre-based programs in terms of cost-effectiveness.

### Limitations

None of the 16 articles met all of Drummond’s 10-item checklist (Table 2). Articles were dated as early as 1985 and as recently as 2009, with 13 of the articles published in the last 10 years. Majority of the studies collected and analyzed only direct medical costs. Few studies considered sensitivity analysis to account for uncertainty in costs and consequences. Although none of the foreign-language articles were included, none met the inclusion criteria for review. Publication bias cannot be excluded as almost all the economic evaluations demonstrated cost-effectiveness.

## Conclusions

In conclusion, evidence exists that supports the inclusion of supervised outpatient centre-based or home-based cardiac rehabilitation compared to no cardiac rehabilitation in patients after myocardial infarction, revascularization surgery or percutaneous coronary interventions, as well as those with chronic heart failure. Based on the reviewed articles, it would appear that the costs and outcomes of home- versus supervised centre-based cardiac rehabilitation were no different. Therefore the choice of the mode of delivery (home- versus centre-based) should be left to purchasers and patients. To the policy decision makers, there could be possible economic advantage of home-based program over centre-based ones. However, the details of what constitute “home-based program” are important.

## Competing interests

The authors declare that they have no competing interests.

## Authors’ contributions

WPW and JF performed article search and screened the abstracts. KHP resolved any disagreements arising from the primary reviewers’ interpretations of the articles and provided input to the manuscript. JL provided input to the interpretation of the review and discussion. All authors read and approved the final manuscript.

## Pre-publication history

The pre-publication history for this paper can be accessed here:

http://www.biomedcentral.com/1472-6963/12/243/prepub

## Supplementary Material

Additional file 1 **Appendix 1. Quality assessment with the 10-item Drummond checklist **[11]**.** Click here for file

Additional file 2 Appendix 1. Summary of Studies on Economic Evaluations of Cardiac Rehabilitation (CR). Click here for file

## References

[B1] Yusuf S Reddy S Ounpuu S Anand S Global burden of cardiovascular diseases: part I: general considerations, the epidemiologic transition, risk factors, and impact of urbanization Circulation 2001 104 22 2746 2753 10.1161/hc4601.099487 11723030

[B2] Levenson JW Skerrett PJ Gaziano JM Reducing the global burden of cardiovascular disease: the role of risk factors Prev Cardiol 2002 5 4 188 199 10.1111/j.1520-037X.2002.00564.x 12417828

[B3] Wenger NK Froelicher ES Smith LK Ades PA Berra K Blumenthal JA Certo CME Dattilo AM Davis D DeBusk RF Cardiac Rehabilitation. Clinical Practice Guideline No. 17 1995 U.S. Department of Health and Human Services, Public Health Service, Agency for Health Care Policy and Research and the National Heart, Lung, and Blood Institute, Rockville, MD 8595435

[B4] Balady GJ Williams MA Ades PA Bittner V Comoss P Foody JM Franklin B Sanderson B Southard D Core components of cardiac rehabilitation/secondary prevention programs: 2007 update: a scientific statement from the American Heart Association Exercise, Cardiac Rehabilitation, and Prevention Committee, the Council on Clinical Cardiology; the Councils on Cardiovascular Nursing, Epidemiology and Prevention, and Nutrition, Physical Activity, and Metabolism; and the American Association of Cardiovascular and Pulmonary Rehabilitation Circulation 2007 115 20 2675 2682 10.1161/CIRCULATIONAHA.106.180945 17513578

[B5] Dafoe W Arthur H Stokes H Morrin L Beaton L Universal access: but when? Treating the right patient at the right time: access to cardiac rehabilitation Can J Cardiol 2006 22 11 905 911 10.1016/S0828-282X(06)70309-9 16971975 PMC2570237

[B6] O'Connor GT Buring JE Yusuf S Goldhaber SZ Olmstead EM Paffenbarger RS Hennekens CH An overview of randomized trials of rehabilitation with exercise after myocardial infarction Circulation 1989 80 2 234 244 10.1161/01.CIR.80.2.234 2665973

[B7] Oldridge NB Guyatt GH Fischer ME Rimm AA Cardiac rehabilitation after myocardial infarction. Combined experience of randomized clinical trials JAMA 1988 260 7 945 950 10.1001/jama.1988.03410070073031 3398199

[B8] Taylor RS Brown A Ebrahim S Jolliffe J Noorani H Rees K Skidmore B Stone JA Thompson DR Oldridge N Exercise-based rehabilitation for patients with coronary heart disease: systematic review and meta-analysis of randomized controlled trials Am J Med 2004 116 10 682 692 10.1016/j.amjmed.2004.01.009 15121495

[B9] Heran BS Chen JM Ebrahim S Moxham T Oldridge N Rees K Thompson DR Taylor RS Exercise-based cardiac rehabilitation for coronary heart disease Cochrane Database Syst Rev 2011 7 CD001800 21735386 10.1002/14651858.CD001800.pub2 PMC4229995

[B10] Papadakis S Oldridge NB Coyle D Mayhew A Reid RD Beaton L Dafoe WA Angus D Economic evaluation of cardiac rehabilitation: a systematic review Eur J Cardiovasc Prev Rehabil 2005 12 6 513 520 16319539 10.1097/01.hjr.0000186624.60486.e8

[B11] Drummond MF Sculpher MJ Torrance GW O'Brien BJ Stoddart GL Methods for the economic evaluation of health care programmes 2005 3 Oxford University Press, New York

[B12] Levin LA Perk J Hedback B Cardiac rehabilitation–a cost analysis J Intern Med 1991 230 5 427 434 10.1111/j.1365-2796.1991.tb00468.x 1940778

[B13] Ades PA Huang D Weaver SO Cardiac rehabilitation participation predicts lower rehospitalization costs Am Heart J 1992 123 4 Pt 1 916 921 1550000 10.1016/0002-8703(92)90696-s

[B14] Oldridge N Furlong W Feeny D Torrance G Guyatt G Crowe J Jones N Economic evaluation of cardiac rehabilitation soon after acute myocardial infarction Am J Cardiol 1993 72 2 154 161 10.1016/0002-9149(93)90152-3 8328376

[B15] Ades PA Pashkow FJ Nestor JR Cost-effectiveness of cardiac rehabilitation after myocardial infarction J Cardiopulm Rehabil 1997 17 4 222 231 10.1097/00008483-199707000-00002 9271765

[B16] Georgiou D Chen Y Appadoo S Belardinelli R Greene R Parides MK Glied S Cost-effectiveness analysis of long-term moderate exercise training in chronic heart failure Am J Cardiol 2001 87 8 984 988 A984 10.1016/S0002-9149(01)01434-5 11305991

[B17] Marchionni N Fattirolli F Fumagalli S Oldridge N Del Lungo F Morosi L Burgisser C Masotti G Improved exercise tolerance and quality of life with cardiac rehabilitation of older patients after myocardial infarction: results of a randomized, controlled trial Circulation 2003 107 17 2201 2206 10.1161/01.CIR.0000066322.21016.4A 12707240

[B18] Yu CM Lau CP Chau J McGhee S Kong SL Cheung BM Li LS A short course of cardiac rehabilitation programme is highly cost effective in improving long-term quality of life in patients with recent myocardial infarction or percutaneous coronary intervention Arch Phys Med Rehabil 2004 85 12 1915 1922 10.1016/j.apmr.2004.05.010 15605326

[B19] Huang Y Zhang R Culler SD Kutner NG Costs and effectiveness of cardiac rehabilitation for dialysis patients following coronary bypass Kidney Int 2008 74 8 1079 1084 10.1038/ki.2008.381 18650790 PMC2777679

[B20] Dendale P Hansen D Berger J Lamotte M Long-term cost-benefit ratio of cardiac rehabilitation after percutaneous coronary intervention Acta Cardiol 2008 63 4 451 456 10.2143/AC.63.4.2033043 18795582

[B21] DeBusk RF Haskell WL Miller NH Berra K Taylor CB Berger WE Lew H Medically directed at-home rehabilitation soon after clinically uncomplicated acute myocardial infarction: a new model for patient care Am J Cardiol 1985 55 4 251 257 10.1016/0002-9149(85)90355-8 3969859

[B22] Lowensteyn I Coupal L Zowall H Grover SA The cost-effectiveness of exercise training for the primary and secondary prevention of cardiovascular disease J Cardiopulm Rehabil 2000 20 3 147 155 10.1097/00008483-200005000-00002 10860196

[B23] Carlson JJ Johnson JA Franklin BA VanderLaan RL Program participation, exercise adherence, cardiovascular outcomes, and program cost of traditional versus modified cardiac rehabilitation Am J Cardiol 2000 86 1 17 23 10.1016/S0002-9149(00)00822-5 10867086

[B24] Collins L Scuffham P Gargett S Cost-analysis of gym-based versus home-based cardiac rehabilitation programs Aust Health Rev 2001 24 1 51 61 10.1071/AH010051 11357742

[B25] Reid RD Dafoe WA Morrin L Mayhew A Papadakis S Beaton L Oldridge NB Coyle D Wells GA Impact of program duration and contact frequency on efficacy and cost of cardiac rehabilitation: results of a randomized trial Am Heart J 2005 149 5 862 868 10.1016/j.ahj.2004.09.029 15894969

[B26] Taylor RS Watt A Dalal HM Evans PH Campbell JL Read KL Mourant AJ Wingham J Thompson DR Pereira Gray DJ Home-based cardiac rehabilitation versus hospital-based rehabilitation: a cost effectiveness analysis Int J Cardiol 2007 119 2 196 201 10.1016/j.ijcard.2006.07.218 17084927

[B27] Papadakis S Reid RD Coyle D Beaton L Angus D Oldridge N Cost-effectiveness of cardiac rehabilitation program delivery models in patients at varying cardiac risk, reason for referral, and sex Eur J Cardiovasc Prev Rehabil 2008 15 3 347 353 10.1097/HJR.0b013e3282f5ffab 18525392

[B28] Jolly K Lip GY Taylor RS Raftery J Mant J Lane D Greenfield S Stevens A The Birmingham Rehabilitation Uptake Maximisation study (BRUM): a randomised controlled trial comparing home-based with centre-based cardiac rehabilitation Heart 2009 95 1 36 42 18332063 10.1136/hrt.2007.127209

[B29] Schweikert B Hahmann H Steinacker JM Imhof A Muche R Koenig W Liu Y Leidl R Intervention study shows outpatient cardiac rehabilitation to be economically at least as attractive as inpatient rehabilitation Clin Res Cardiol 2009 98 12 787 795 10.1007/s00392-009-0081-6 19821135

[B30] Wheeler JR Janz NK Dodge JA Can a disease self-management program reduce health care costs? The case of older women with heart disease Med Care 2003 41 6 706 715 12773836 10.1097/01.MLR.0000065128.72148.D7

[B31] Southard BH Southard DR Nuckolls J Clinical trial of an Internet-based case management system for secondary prevention of heart disease J Cardiopulm Rehabil 2003 23 5 341 348 10.1097/00008483-200309000-00003 14512778

[B32] Salvetti XM Oliveira JA Servantes DM Vincenzo de Paola AA How much do the benefits cost? Effects of a home-based training programme on cardiovascular fitness, quality of life, programme cost and adherence for patients with coronary disease Clin Rehabil 2008 22 10–11 987 996 18955430 10.1177/0269215508093331

[B33] Hall JP Wiseman VL King MT Ross DL Kovoor P Zecchin RP Moir FM Denniss AR Economic evaluation of a randomised trial of early return to normal activities versus cardiac rehabilitation after acute myocardial infarction Heart Lung Circ 2002 11 1 10 18 10.1046/j.1444-2892.2002.00105.x 16352063

