# Supplementary material for: A systematic review of economic evaluations of cardiac rehabilitation
Source: BMC Health Serv Res. 2012 Aug 8;12:243. doi: 10.1186/1472-6963-12-243 (PMC3465180; doi:10.1186/1472-6963-12-243)
Supplement: Additional file 1 — Appendix 1. Quality assessment with the 10-item Drummond checklist [11]. [file 1472-6963-12-243-S1.doc]

**Appendix 1: Quality assessment with the 10-item Drummond checklist (11)**.

| **Checklist** | | | | | | | | | | |
| --- | --- | --- | --- | --- | --- | --- | --- | --- | --- | --- |
| 1. Was a well-defined question posed in answerable form? | | | | | | | | | | |
| 1. Was a comprehensive description of the competing alternatives given (i.e. can you tell who did what to whom, where, and how often)? | | | | | | | | | | |
| 1. Was the effectiveness of the programme or services established? | | | | | | | | | | |
| 1. Were all the important and relevant costs and consequences for each alternative identified? | | | | | | | | | | |
| 1. Were costs and consequences measured accurately in appropriate physical units (e.g. hours of nursing time, number of physician visits, lost work-days, gained life years)? | | | | | | | | | | |
| 1. Were the cost and consequences valued credibly? | | | | | | | | | | |
| 1. Were costs and consequences adjusted for differential timing? | | | | | | | | | | |
| 1. Was an incremental analysis of costs and consequences of alternatives performed? | | | | | | | | | | |
| 1. Was allowance made for uncertainty in the estimates of costs and consequences? | | | | | | | | | | |
| 1. Did the presentation and discussion of study results include all issues of concern to users? | | | | | | | | | | |
| First author [reference] | 1 | 2 | 3 | 4 | 5 | 6 | 7 | 8 | 9 | 10 |
| Levin [12] | 🗶 | ✓ | ✓ | 🗶 | ✓ | ⁰ | ✓ | ✓ | ✓ | ✓ |
| Ades [13] | 🗶 | ✓ | ✓ | 🗶 | ✓ | ⁰ | 🗶 | ✓ | ✓ | ✓ |
| Oldridge [14] | ✓ | ✓ | ✓ | ✓ | ✓ | ⁰ | ✓ | ✓ | ✓ | ✓ |
| Ades [15] | ✓ | ✓ | ✓ | 🗶 | ✓ | ⁰ | ✓ | ✓ | ✓ | ✓ |
| Georgiou [16] | ✓ | ✓ | ✓ | 🗶 | 🗶 | ✓ | ✓ | ✓ | ✓ | 🗶 |
| Marchionni [17] | 🗶 | ✓ | ✓ | 🗶 | 🗶 | ✓ | 🗶 | 🗶 | 🗶 | 🗶 |
| Yu [18] | ✓ | ✓ | ✓ | 🗶 | 🗶 | ✓ | 🗶 | ✓ | 🗶 | ✓ |
| Huang [19] | ✓ | 🗶 | ✓ | 🗶 | ✓ | ✓ | 🗶 | ✓ | 🗶 | ✓ |
| Dendale [20] | ✓ | ✓ | ✓ | 🗶 | 🗶 | ⁰ | 🗶 | 🗶 | 🗶 | 🗶 |
| Debusk [21] | 🗶 | ✓ | ✓ | 🗶 | 🗶 | ⁰ | 🗶 | 🗶 | 🗶 | 🗶 |
| Lowensteyn [22] | 🗶 | ✓ | ✓ | 🗶 | 🗶 | 🗶 | ✓ | ✓ | ✓ | ✓ |
| Carlson [23] | 🗶 | ✓ | ✓ | 🗶 | 🗶 | 🗶 | ✓ | ✓ | ✓ | 🗶 |
| Collins [24] | 🗶 | ✓ | ✓ | 🗶 | 🗶 | 🗶 | ✓ | 🗶 | ✓ | 🗶 |
| Hall [33] | 🗶 | ✓ | ✓ | ✓ | ✓ | ⁰ | 🗶 | 🗶 | 🗶 | ✓ |
| Reid [25] | 🗶 | ✓ | ✓ | 🗶 | 🗶 | ⁰ | 🗶 | ✓ | 🗶 | 🗶 |
| Taylor [26] | ✓ | ✓ | ✓ | 🗶 | ✓ | ✓ | 🗶 | ✓ | 🗶 | ✓ |
| Papadakis [27] | ✓ | ✓ | ✓ | 🗶 | ✓ | ✓ | 🗶 | ✓ | ✓ | ✓ |
| Jolly [28] | ✓ | ✓ | ✓ | 🗶 | ✓ | ✓ | 🗶 | ✓ | 🗶 | ✓ |
| Schweikert [29] | 🗶 | ✓ | ✓ | 🗶 | ✓ | ⁰ | 🗶 | ✓ | ✓ | ✓ |
| Wheeler [30] | ✓ | ✓ | ✓ | 🗶 | ✓ | 🗶 | 🗶 | 🗶 | 🗶 | 🗶 |
| Southard [31] | 🗶 | ✓ | ✓ | 🗶 | ✓ | ✓ | ✓ | 🗶 | 🗶 | ✓ |
| Salvetti [32] | 🗶 | ✓ | ✓ | 🗶 | ✓ | ⁰ | 🗶 | 🗶 | 🗶 | ✓ |

yes ✓, no 🗶, cannot tell ⁰
