# Supplementary material for: A systematic review of economic evaluations of cardiac rehabilitation
Source: BMC Health Serv Res. 2012 Aug 8;12:243. doi: 10.1186/1472-6963-12-243 (PMC3465180; doi:10.1186/1472-6963-12-243)
Supplement: Additional file 2 — Appendix 1. Summary of Studies on Economic Evaluations of Cardiac Rehabilitation (CR). [file 1472-6963-12-243-S2.doc]

Appendix 2. Summary of Studies on Economic Evaluations of Cardiac Rehabilitation (CR).

| Author (year) Country  Perspective | Intervention | Comparator | Patient population | Study Type  Time Frame  Discounting | Clinical evidence (source, results) | Costs, (currency,  price year),  Outcome measures | Results  Conclusion |
| --- | --- | --- | --- | --- | --- | --- | --- |
| *Supervised Centre-Based CR* versus *No CR* | | | | | | | |
| Levin et al (1991)  Sweden  Societal | CR – follow-up at post-MI clinic, health education, outpatient physical training | No CR | N=305 post MI, <65 years old | CCA  5 years  Discounting not applicable | Non-randomized trial, patients after CR returned to work more frequently with less costs due to loss of productivity | Direct medical costs (Swedish kroners, price year unknown),  Cost per patient | SEK73,500 less per patient cost in CR than in No-CR group.  CR was cost-saving. |
| Ades et al (1992)  USA  Patients and payers | CR – 12 weeks of four hours of aerobic exercise training and risk factor management | No CR | N=580 post MI/CABG | CCA  1-46 months (mean 21 months)  Discounting not applicable | Non-randomized trial, lower re-hospitalization rates among patients who participated in CR | Re-hospitalization costs (US Dollars, price year unknown),  Hospitalization costs per patient | USD739 less in hospitalization costs per patient in CR than in No-CR group.  CR was cost-saving. |
| Oldridge et al (1993)  Canada  Societal | CR – 8 weeks (16 sessions) of supervised exercise training, group behavioural and risk factor management | No CR | N=201 moderately anxious and depressed patients post MI | CEA/CUA  12 months for primary data; 36 months for modelled data  5% | Data from RCT and systematic reviews, patients in CR group gained more QALYs than those in No-CR group | Direct and indirect medical costs (US Dollars, 1991),  Cost per year of life saved and cost per QALY gained and | $21,800 per life-year gained; $9,200 per QALY gained at 1 year and $6,800 per QALY gained at 3 year.  CR was cost-effective. |
| Ades et al (1997)  USA  Patients and payers | CR – 12 weeks of exercise training thrice weekly | No CR | Not applicable | CEA  15 years  Discounting not applicable | Economical modelling | Direct medical costs (US Dollars, 1995),  Cost per year of life saved | $2,130 per year of life saved for 1985, but $4,950 for 1995.  CR was still cost-effective, though less so over a 10-year period. |
| Georgiou et al (2001)  USA  Societal | CR – thrice weekly over 8 weeks then twice weekly over 12 months | No CR | N=99 patients with heart failure aged 55-64 years and NYHA III | CEA  14 months for primary data and 10 years for modelled data  Discounting not stated | Data from RCT and modelling, patients in CR group incurred 19% reduction in hospitalization rates | Direct medical costs (US Dollars, 1999),  Incremental cost-effectiveness ratio (ICER) | ICER = $1,773 per life year saved.  CR was dominant strategy. |
| Marchionni et al (2003)  Italy  Government or health care providers | CR – 40 sessions of aerobic and stretching exercises; risk factor counselling twice per week; monthly support group | No CR | N=158 patients with MI | CCA  14 months  Discounting not applicable | RCT, patients in CR improved in exercise tolerance and quality of life across different age groups for both gender | Direct medical costs (US Dollars, 2000),  Cost per program | $21,298 per patient for CR group; $12,433 per patient for No-CR group.  CR was cost-effective. |
| Yu et al (2004)  Hong Kong  Government | CR – four phases: 1) 7-14-day inpatient walking program; 2) 8 weeks of out-patient twice weekly education and aerobic exercise; 3) 6 months of community-based home exercise program; 4) long-term maintenance program till end of follow-up period | No CR | N=204 patients after MI or PCI | CUA  2 years  Discounting not applicable | RCT, patients in CR group needed PCI less with net gain in quality of life | Direct medical costs, (USD, price year not mentioned),  Incremental cost-utility ratio (ICUR) | ICUR, -$650 per QALY.  CR was a dominant strategy. |
| Huang et al (2008)  USA  Government | CR – 36 exercise sessions thrice weekly over 12 weeks; details not described by authors | No CR | N=4,324 patients with end-stage renal failure and on chronic haemo-dialysis, after CABG | CEA  Up to 42 months (average 20.3 months)  Discounting not applicable | Observational data from Medicare database, patients stratified by propensity scores, patients from CR group had $2,904 (95% CI: -7,028, 11,940) greater Medicare expenditure but 76 days (95% CI: 22, 129) longer cumulative lifetime | Medicare expenditure, (USD, 1998),  Incremental cost-effectiveness ratio (ICER) | ICER $13,887 per year of life saved.  CR was highly cost-effective. |
| Dendale et al (2008)  Belgium  Health care payers | CR – At least 24 supervised aerobic exercise sessions thrice weekly over at least 3 months; psychological counselling; dietary advice; smoking cessation program (8 sessions) | No CR | N=213 patients after PCI | CEA  4.5 years  Discounting not applicable | Non-randomized 2-group, patients in CR group had reduction of hospitalization (45% vs 75%), revascularization (7% vs 17%) and average event per patient (0.93 vs 1.52) compared to No-CR group | Direct medical costs, (Euro, price year not stated),  Health care cost per patient | 4,862 Euro per patient for CR group compared to 5,498 Euro per patient for No-CR group.  CR was cost-saving. |
| *Home-Based CR* versus *Supervised Centre-Based CR* | | | | | | | |
| Debusk et al (1985)  USA  Patients | Home-Based – exercise program with loan of cycle or given walking program; home monitoring via portable heart rate monitors and ECG | Centre-Based – 8-26 weeks of thrice weekly supervised exercise training | N=127 patients post MI | CMA  2 years  Discounting not applicable | RCT, no significant difference in exercise capacity and cardiac complications during exercise between home- and centre-based CR groups | Direct medical costs, (US Dollars, 1985),  Health care cost per patient | $328 per patient for home-based CR; $720 per patient for centre-based CR.  Home-Based CR was cost-saving. |
| Carlson et al (2000)  USA  Health care payers | Home-Based –38 sessions of CR at centre, initially more visits and then less with more home-based self-management | Centre-Based – 42 sessions of CR | N=80 patients post MI and low to moderate risk | CMA  6 months  Discounting not applicable | RCT, no significant difference in physiologic indices such as body mass index, resting heart rate and functional capacity | CR costs, (US Dollars, price year not stated),  Cost per patient | $1,519 per patient from home-based group and $2,349 per patient from centre-based group.  Home-Based CR was cost-saving. |
| Collins et al (2001)  Australia  Patients and payers | Home-Based – 12 months, case manager visits, self-monitoring, walking program, phone calls, home visits, education materials and videotapes | Centre-Based – gym-based, supervision, 8 weeks, multidisciplinary team management, group education and support sessions, educational materials | n=94 patients in primary data but details not described | CMA  12 months for modelling and 18 months for primary data  27% over 5 years | Retrospective data analysis on 94 patients and modelling based on selective review; assumption of the model was that clinical effects both modes of delivery of CR were similar | CR costs, (Australian Dollars, price year not stated),  Cost per patient | $1,169 per patient for home-based CR and $1,933 per patient for centre-based CR.  Home-Based CR was cost-saving. |
| Hall et al (2002)  Australia  Societal | Home-Based – early return to normal activities at 2 weeks after MI, education about heart disease risk factor, counselling and home walking program | Centre-Based – low-level exercise program, counselling on group behavioural and risk factor management plus education about heart disease risk factor, home walking program | N=127 low-risk patients after MI, <75 years | CMA  12 months  Discounting not applicable | RCT, no significant difference in clinical and quality of life outcomes for both groups | CR costs, (Australian Dollars, 1999),  Cost per patient | $28.12 per patient per exercise session and $393.68 per patient treated in hospital for the centre-based group.  Home-Based CR was cost-saving. |
| Lowensteyn et al (2000)  Canada  Societal | Home-Based – walking program | Centre-Based – group supervised program | N=1,486 patients with cardiovascular disease (CVD) | CEA  Time frame not stated  3% | Primary data from Canadian Heart Health Survey 1986-1992; modelling up to age 102 years | CR costs, (US Dollars, 1996),  Cost per year of life saved | <$12,000 per year of life saved for home-based CR and <$20,000 per year of life saved for all men and older women with CVD.  Home-Based CR was more cost-effective than Centre-Based CR. |
| Marchionni et al (2003)  Italy  Government or health care providers | Home-Based – 4 to 8 supervised sessions in the centre and then continued at home with wristwatch digital pulse monitor, cycle ergometer, and a log book; home visits by PT every other week or as necessary, plus cardiovascular risk factor management counseling at each in-hospital session and monthly family-oriented support group | Centre-Based – 40 sessions of aerobic and stretching exercises; risk factor counseling twice per week; monthly support group | N=153 patients with MI | CMA  14 months  Discounting not applicable | RCT, no significant difference in improvements between two groups | Direct medical costs (US Dollars, 2000),  Cost per program | $13,246 per patient for Home-based group; $21,298 per patient for centre-based-CR.  Home-Based CR was cost-saving compared to Centre-Based CR. |
| Reid et al (2005)  Canada  Health system | Home-Based – 2 case manager visits, telephone contacts, physician visit and supervised exercise classes were held once per week for 14 weeks, once every 2 weeks for 14 weeks, and once every 4 weeks for 24 weeks. Case manager provided risk factor modifications; physician provided information on disease and complications. | Centre-Based – 2 case manager visits, telephone contact, physician visit and more frequent supervised exercise classes held twice weekly for a 13½-week period. Case manager provided risk factor modifications; physician provided information on disease and complications. | N=392 patients with coronary artery disease | CCA  24 months  Discounting not applicable | RCT, both groups showed improvement in clinical and health indices, although no statistically significant difference between groups | Direct medical costs, (US Dollars, 2004),  Cost per patient | $5,267 per patient for home-based group and $5,132 per patient for centre-based group.  No difference between Home- and Centre-Based in terms of cost-savings. |
| Taylor et al (2007)  UK  Societal | Home-Based – Heart Manual, 3 home visits, telephone contacts by rehab nurse, additional visits as required. Heart Manual contained exercise, relaxation, education and lifestyle information | Centre-Based – 9 sessions at weekly intervals, 12 sessions over 8 weeks and 24 individualised sessions over 12 weeks. CR sessions included exercise, relaxation, education and lifestyle counselling | N=80 patients with MI | CUA  9 months  Discounting not applicable | RCT, no significant difference between both groups in terms of health and clinical indices | Direct medical costs, (Sterling pounds, 2002-3),  Incremental cost-utility ratio | ICUR -₤644 per QALY in favour of Centre-Based CR but not significantly different; Home-Based CR cost only ₤30 less than Centre-Based CR because of high costs involved in cardiac investigations and surgery.  Neither mode of delivery of CR was more cost-effective than the other. |
| Papadakis et al (2008)  Canada  Health system | Home-Based – 33 sessions over 12 months (ranging from weekly to monthly), including exercise training, education classes, behaviour modification and physician consults | Centre-Based – 33 sessions over 3 months, twice weekly, including exercise training, education classes, behaviour modification and physician consults | N=392 patients with coronary artery disease | CUA  24 months  Discounting not applicable | RCT, patients in centre-based CR had more QALY gained than those in home-based CR | Direct medical costs, (US Dollars, 2004),  Incremental cost-utility ratio ICUR) | ICUR $11,400 per QALY; sub-group analysis showed centre-based CR was more cost-effective and less expensive for high-risk patients and men but home-based CR was more cost-effective for women and those who underwent PCI.  Centre-Based CR was dominant strategy. |
| Jolly et al (2009)  UK  Societal | Home-Based – Heart Manual, 3 home visits, telephone contacts by rehab nurse, additional visits as required. Heart Manual contained exercise, relaxation, education and lifestyle information | Centre-Based – 9 sessions at weekly intervals, 12 sessions over 8 weeks and 24 individualised sessions over 12 weeks. CR sessions included exercise, relaxation, education and lifestyle counselling | N=525 patients with MI or CABG | CMA  12 months  Discounting not applicable | RCT, no significant difference in health and clinical indices between both groups | Direct medical costs, (Sterling pounds, 2002-3),  Cost per patient | ₤807 (95% CI 684, 930) per patient for home-based group and ₤896 (95%CI 745, 1047) per patient for centre-based group. Cost to the National Health Services (government) was ₤198 (95% CI 189, 208) per patient for home-based group, but ₤157 (95% CI 139, 175) per patient for centre-based group.  Neither group was more cost-saving to patients. Home-Based cost slightly more for the government. |
| *Inpatient CR* versus *Outpatient CR* | | | | | | | |
| Schweikert et al (2009)  Germany  Societal | Inpatient – average 6 h per day, including exercise training, education, relaxation and dietary counselling; patient stayed in hospital | Outpatient – average 6 h per day with similar contents; patient left late afternoon | N=147 patients with MI | CEA/CUA  12 months  Discounting not applicable | Case-control design, no significant difference in quality of life between both groups | Direct medical costs, (Euro, 2006),  Incremental cost-effectiveness ratio (ICER) | ICER -165,276 Euro per QALY (95% CI 14,401, -34,414). Outpatient CR was the dominant strategy although statistically insignificant. Direct rehabilitation costs between both groups were not significantly different; total direct medical costs were.  Outpatient CR was preferred economically. |
| *Home-Based CR* versus *No CR* | | | | | | | |
| Wheeler (2003)  USA  Patients or payers | Home-Based - “Women take PRIDE” self-management program – consisting of 49 sessions meeting in groups of 6-8 for 2½ hours during 4 consecutive weeks facilitated by health educator and peer leader, patient given workbook, videotape and self-monitoring tool such as pedometer. | No CR | N=452 women >/= 60 years with MI, heart failure, valvular diseases, etc. | CCA  3 years  Discounting not applicable | RCT, home-based participants had 46% fewer inpatient days than controls (P<0.05), although no significant difference between both groups in terms of emergency department utilization | Hospitalization and emergency room visit costs, (US Dollars, 2000),  Cost-saving per person | Home-based participants experienced 49% lower inpatient costs (P <0.10) than the control group, resulted in a cost saving of $3200 per patient per year.  Home-Based CR was cost-saving compared to no CR. |
| Southard et al (2003)  USA  Patients | Home-Based – internet-based program involving logging on to the site at least once a week for 30 minutes, messaging with a case manager, completing education modules (with self-tests), and entering data (eg, number of minutes of exercise, blood pressure measurements) into progress graphs; small rewards incentives for active participation. | No CR | N=104 patients with MI, CABG and heart failure | CCA/CBA  6 months  Discounting not applicable | RCT, no significant difference in physiologic and clinical indices | Cost of professional time involved in internet program, cardiovascular-related emergency room visits and hospitalization, (US Dollars, price year not stated),  Cost per person and return on investment | Home-based group cost $1,418 less than No-CR group with 213% return on investment.  Home-Based CR was cost-saving compared to no CR. |
| Marchionni et al (2003)  Italy  Government or health care providers | Home-Based – 4 to 8 supervised sessions in the centre and then continued at home with wristwatch digital pulse monitor, cycle ergometer, and a log book; home visits by PT every other week or as necessary, plus cardiovascular risk factor management counseling at each in-hospital session and monthly family-oriented support group | No CR | N=153 patients with MI | CCA  14 months  Discounting not applicable | RCT, patients in home-based CR group improved in exercise tolerance and quality of life across different age | Direct medical costs (US Dollars, 2000),  Cost per program | $13,246 per patient for Home-based group; $12,433 per patient for No-CR group.  Home-Based CR was cost-effective. |
| Salvetti et al (2008)  Brazil  Health providers | Home-Based – 2 sessions with PT, and then given an exercise log to follow at home, doctors called every 2 months to check on exercise adherence. | No CR | N=39 with coronary artery disease with NYHA I & II | CCA  3 months  Discounting not applicable | RCT, patients in the CR group had significant improvement in all 8 domains of the SF-36. However, the control group showed improvement in only 3 domains and decline in the other 5 domains. | Direct medical costs, (US Dollars, price year not stated).  Cost per patient | Additional $502.71 per patient for the home-based CR group.  Home-Based CR was low-cost and affordable. |

CR, cardiac rehabilitation. RCT, randomized controlled trial. PT, physical therapy/therapist. NYHA, New York Heart Association classification. MI, myocardial infarction. CABG, coronary artery bypass graft surgery. PCI, percutaneous coronary intervention. CCA, cost-consequences analysis. CEA, cost-effectiveness analysis. CUA, cost-utility analysis. CBA, cost-benefit analysis. CMA, cost-minimization analysis. QALY, quality-adjusted life-years. ICER, incremental cost-effectiveness ratio. ICUR, incremental cost-utility ratio.
